# Intradetrusor onabotulinumtoxinA injections are significantly more efficacious than oral oxybutynin for treatment of neurogenic detrusor overactivity: results of a randomized, controlled, 24-week trial

**DOI:** 10.1590/S1679-45082018AO4207

**Published:** 2018-08-01

**Authors:** Rúiter Silva Ferreira, Carlos Arturo Levi D’Ancona, Matthias Oelke, Maurício Rassi Carneiro

**Affiliations:** 1 Universidade Estadual de Campinas, Campinas, SP, Brazil; Centro de Reabilitação e Readaptação Dr. Henrique Santillo, Goiânia, GO, Brazil.; 2 Universidade Estadual de Campinas, Campinas, SP, Brazil.; 3 Department of Urology, Academic Hospital Maastricht, University of Maastricht, Maastricht, The Netherlands.; 4 Centro de Reabilitação e Readaptação Dr. Henrique Santillo, Goiânia, GO, Brazil.

**Keywords:** Mandelic acids, Botulinum toxins, type A, Urinary bladder, neurogenic, Spinal cord injury, Urodynamics, Quality of life, Ácidos mandélicos, Toxinas botulínicas Tipo A, Bexiga urinária neurogênica, Traumatismos da medula espinal, Urodinâmica, Qualidade de vida

## Abstract

**Objective:**

To prospectively compare the results of intradetrusor onabotulinumtoxinA injections and oral oxybutynin for urinary continence, urodynamic parameters and quality of life in patients with neurogenic detrusor overactivity due to spinal cord injury.

**Methods:**

Adult patients under intermittent catheterization were randomized 1:1 to receive one injection of onabotulinumtoxinA 300U or oxybutynin 5mg, *per oris*, three times/day. Primary study endpoint was change in urinary incontinence episodes/24 hours and secondary study endpoints were maximum cystometric capacity, maximum detrusor pressure, bladder compliance and quality of life before randomization and at week 24.

**Results:**

Sixty-eight patients participated in the trial. Significant improvements in urinary incontinence per 24 hours, all investigated urodynamic parameters and quality of life were observed in both groups. Compared with oral oxybutynin, onabotulinumtoxinA was significantly more efficacious for all parameters investigated. Non-response to treatment was higher for oral oxybutynin (23.5%) than onabotulinumtoxinA (11.8%). Dry mouth was the most common adverse in patients with oral oxybutynin (72%) and transient macroscopic hematuria in patients with onabotulinumtoxinA (28%). Only one patient with oral oxybutynin dropped out the study because of adverse effects.

**Conclusion:**

The comparison of the two study drugs showed that onabotulinumtoxinA was significantly more efficacious than oral oxybutynin with regard to continence, urodynamic parameters and quality of life. Clinicaltrials.gov: NCT:01477736.

## RESUMO

**Objetivo:**

Comparar prospectivamente os resultados de injeções intradetrusoras de onabotulinumtoxinA e oxibutinina oral em pacientes com hiperatividade neurogênica do detrusor devido à lesão da medula espinhal, para avaliar a continência urinária, os parâmetros urodinâmicos e a qualidade de vida.

**Métodos:**

Pacientes adultos em cateterismo intermitente foram randomizados 1:1 para tratamento com uma injeção de onabotulinumtoxinA 300U ou oxibutinina 5mg via oral, três vezes por dia. O desfecho primário foi alteração nos episódios de incontinência urinária em 24 horas, e os secundários foram capacidade cistométrica máxima, pressão máxima do detrusor, complacência vesical e qualidade de vida antes da randomização e na 24ª semana.

**Resultados:**

Participaram do estudo 68 pacientes. Observou-se melhora significativa na incontinência urinária por 24 horas em todos os parâmetros urodinâmicos investigados e na qualidade de vida em ambos os grupos. Em comparação com a oxibutinina oral, a onabotulinumtoxinA foi significativamente mais eficaz para todos os parâmetros investigados. A falha no tratamento foi maior para oxibutinina oral (23,5%) em comparação com onabotulinumtoxinA (11,8%). A boca seca foi o evento adverso mais comum em pacientes tratados com oxibutinina oral (72%), e a hematúria macroscópica transitória naqueles tratados com onabotulinumtoxinA (28%). Apenas um paciente tratado com oxibutinina oral interrompeu o estudo por conta dos efeitos adversos.

**Conclusão:**

A comparação dos dois fármacos do estudo mostrou que onabotulinumtoxinA foi significativamente mais eficaz que oxibutinina oral em relação a continência, parâmetros urodinâmicos e qualidade de vida. Clinicaltrials.gov: NCT:01477736.

## INTRODUCTION

Spinal cord injury (SCI) is associated with neurogenic bladder dysfunction and neurogenic detrusor overactivity (NDO). The primary treatment goal is preservation of renal function.^(^ ^1^ ^)^ Patients with neurogenic bladder dysfunction frequently complain about urinary incontinence that may severely affect quality of life.^(^ ^2^ ^)^

Oral antimuscarinics, such as oxybutynin (Oxy), are widely used as first-line treatment of NDO. However, Oxy or other antimuscarinics may be inefficacious in some patients and cause adverse events, such as dry mouth, constipation or blurred vision.^(^ ^3^ ^)^

Intradetrusor injections of onabotulinumtoxinA (OnabotA) have been used as a second-line treatment for patients who do not tolerate or inadequately respond to antimuscarinic agents.^(^ ^4^ ^)^ The effects of OnabotA on the neuromuscular junction have been extensively investigated and consist of inhibition of acetylcholine release and muscle relaxation.^(^ ^5^ ^)^ OnabotA also inhibits other neurotransmitters (*e.g*. adenosine triphosphate) or neuropeptides (*e.g*. substance P),^(^ ^6^ ^)^ and regulates the expression of purinergic receptors and transient receptor potential vanilloid receptor-1 (TRVP1) in afferent bladder wall neurons.^(^ ^7^ ^)^ Intradetrusor OnabotA therapy for NDO improves urodynamic parameters, such as maximum cystometric capacity (MCC), maximum detrusor pressure (Pdet_max_) and bladder compliance, in addition to quality of life.^(^ ^5^ ^,^ ^8^ ^)^ Wu et al.,^(^ ^9^ ^)^ reported the cost-benefit ratio of OnabotA treatment is superior to that of antimuscarinics for NDO. OnabotA has also shown to significantly reduce drug costs associated with urinary tract infections.^(^ ^10^ ^)^

To date, no randomized trial has ever compared oral antimuscarinics with intradetrusor OnabotA injections for treating NDO.

## OBJECTIVE

To prospectively compare the results of oral oxybutynin and intradetrusor onabotulinumtoxinA injections in patients with neurogenic detrusor overacitvity.

## METHODS

### Study design and patient selection

This prospective, randomized and controlled clinical trial was performed in adult patients with SCI and NDO in two centers. The study was initiated in 2010 after approval by the Ethics Committee of the *Faculdade de Ciências Médicas da Universidade Estadual de Campinas*, under number 118/2010, CAAE: 0098.0.146.000-10, and endorsement by the Board of the *Centro de Reabilitação e Readaptação Dr. Henrique Santillo* (CRER). Each patient read and signed the informed consent before enrollment.

Patients were randomized 1:1 by using sealed, opaque, sequentially numbered envelopes and, afterwards, received immediate-release oral Oxy 5mg three-times/day, or one intradetrusor injection of 300U OnabotA. The primary endpoint was to determine the number of incontinence episodes in 24 hours, in a 3-day bladder diary; and the secondary endpoint was to evaluate changes in urodynamic parameters (MCC, Pdet_max_, and bladder compliance) as well as in quality of life, as measured by the Qualiveen questionnaire, between baseline and week 24.

The main inclusion criteria were age >18 years, SCI for at least 12 months, and regular clean intermittent catheterization. Exclusion criteria included pregnancy, a desire to become pregnant during the study period, breastfeeding, use of anticoagulants or known coagulation disorders, neuromuscular transmission disorder, use of any intravesical drug, or previous use of OnabotA. All patients discontinued oral antimuscarinics 7 days before baseline evaluation, and received antibiotics before treatment. Urinary continence was defined as the absence of urinary incontinence in the intervals between catheterizations.^(^ ^1^ ^)^

All patients were first clinically evaluated according to the neurologic scale developed by the American Spinal Injury Association (ASIA).^(^ ^11^ ^)^ Laboratory tests included blood urea nitrogen and serum creatinine concentrations, urinalysis and, if indicated, urine culture and imaging. Patients were asked to keep a 3-day bladder diary, documenting frequency, hours and incontinence episodes between catheterizations. All patients also completed the Qualiveen questionnaire, which is a disease-specific tool developed to evaluate the general and urinary-related impact on quality of life in SCI patients. The validated questionnaire underwent successful cross-cultural adaptation into English and was later translated into Portuguese.^(^ ^12^ ^)^ Urodynamic studies were carried out according to the recommendations of the International Continence Society (ICS).^(^ ^13^ ^,^ ^14^ ^)^

### Patient treatment and follow-up

Intradetrusor injections were performed on the third day of antibiotic course in a surgical setting, when patients were sedated with 2mg/kg of intravenous propofol. Three hundred units of OnabotA (Botox^®^, Allergan, São Paulo, SP, Brazil) were injected into the detrusor with low bladder filling, as described by Schurch et al.^(^ ^8^ ^)^ The dose of 300U was chosen because at the time of recruitment this dose was the most frequently used and recommended for the treatment of NDO.

The bladder diary, urodynamic testing with determination of baseline urodynamic parameters, and quality of life evaluation were performed before randomization, repeated at week 4 and at the end of follow-up period at week 24. Patients were asked to report about any adverse event during the follow-up visits. Treatment non-response was defined when urodynamic parameters did not improve and urinary incontinence was not reduced. Non-responders were offered OnabotA injection therapy after 12 weeks (OnabotA Group) or 24 weeks (Oxy Group).

### Statistical analysis

The study was planned to accept an error of 5% and a power of 80%. The sample size was determined by using the effect size of urodynamic parameters (primary efficacy parameters) of previous studies while the quality of life scores were determined as secondary efficacy parameter. The χ^2^ test and Fisher’s exact test were used to compare categorical variables between the two groups. For comparison of baseline parameters of the two treatment groups, continuous variables were analyzed by the Mann-Whitney U test since groups and parameters were not normally distributed. The Wilcoxon test was applied to compare any intragroup variations in numeric variables between the baseline evaluations and study end. The significance level for the statistical tests was 5%. SAS version 9.1.3 (SAS Institute, USA) was employed for all statistical analyses.

## RESULTS

**Figure 1:**
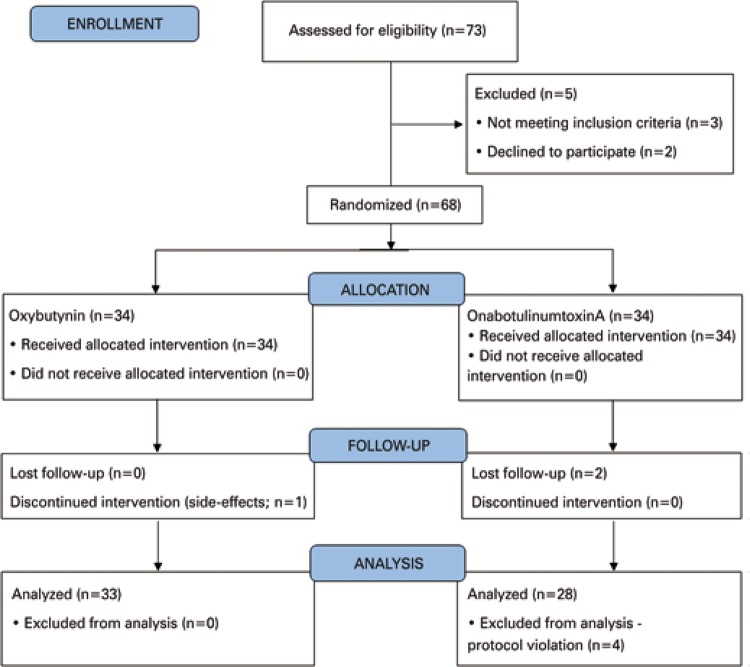
Trial flowchart to demonstrate the number of patients who were enrolled, allocated to oxybutynin or onabotulinumtoxinA, followed-up, and analyzed

A total of 73 patients accepted to participate in this study; however, 5 were excluded for not fulfilling the inclusion criteria or drop out. Therefore, 68 patients participated in the study and were randomly selected to receive Oxy or OnabotA (Figure 1). Seven patients (10.3%) prematurely discontinued the study. Of 61 remaining patients who completed the study, 49 (80.3%) were male. The demographic and baseline data of patients who completed the study are summarized in table 1. No statistically significant differences were found between the two groups.

**Table 1:** Baseline parameters of the oxybutynin and onabotulinumtoxinA treatment groups

	Oxi (n=33)	OnabotA (n=28)	p value
Male/female	26/7	23/5	0.743^*^
Mean age (±SD)	31±8	33±11	0.839^#^
Range	(22-52)	(19-61)	
Mean time SCI (months, ±SD)	25±10	23±8	0.533^#^
Range	(12-61)	(12-47)	
Neurological level			0.956^‡^
T1-T6	23	21	
T7-T12	9	7	
L1	1	0	
Mean incontinence episodes/24h (±SD)	8±1	7±1	0.773^#^
Range	(5-10)	(6-9)	

* χ^2^ test; ^#^ Mann-Whitney test; ^‡^ Fisher’s exact test.

ASIA: American Spinal Injury Association (score); MCC: maximum cystometric capacity; Pdet_max:_ maximum detrusor pressure; QoL: quality of life; SD: standard deviation; SIUP: specific impact of urinary problems.

### Efficacy parameters

Both Oxy and OnabotA treatments resulted in significant improvement in number of incontinence episodes in 24 hours (Table 2). Maximum cystometric capacity and bladder compliance significantly increased, while Pdet_max_ decreased. Both treatments promoted a significant impact on quality of life. There were less adverse effects with OnabotA than with oxy.

**Table 2:** Comparison of the number of incontinence episodes per 24 hours, urodynamic parameters, and quality of life scores in the oxybutynin (Oxy) and onabotulinumtoxinA (OnabotA) treatment groups between baseline and week 24

Treatment	Oxybutynin Group				OnabotulinumtoxinA Group				Inter-group difference (Oxy *versus* OnabotA)
	Mean±SD				Mean±SD				
	Range				Range				
			
Parameters	Baseline	Week 24	Intra-group difference	p value*	Baseline	Week 24	Intra-group difference	p value*	p value^†^
Incontinence episodes per 24hs^‡^	8±1	5±2	-2.3±1.9	<0.001	7±1	1±3	-7.6±3.5	<0.001	<0.001
	(5–10)	(1-10)			(6-9)	(0-11)			
MCC	167±36	293±69	126±62	<0.001	172±33	461±139	289±135	<0.001	<0.001
	(87-210)	(121-410)			(115-225)	(130-600)			
Pdet_max_	79±21	58±19	-21±20	<0.001	79±21	30±27	-49±29	<0.001	<0.001
	(43-121)	(29-110)			(36–114)	(5–105)			
Bladder compliance	14±4	21±4	7±5	<0.001	15±3	40±24	26±24	<0.001	0.006
	(6-21)	(13-31)			(6-20)	(13–120)			
Qualiveen, SIUP	3.2±0.4	3.0±0.5	-0.3±0.3	<0.001	3.4±0.4	1.9±0.7	-1.5±0.7	<0.001	<0.001
	(2.4-4.0)	(2.00-4.00)			(2.45-4.00)	(1.2-4.0)			
Qualiveen, index	-1.0±0.6	-0.9±0.6	0.1±0.3	<0.001	-1.3±0.5	-0.7±0.5	0.6±0.5	<0.001	<0.001
	(-2.0– -0.1)	(-2.0-0.1)			(-2.0– -0.3)	(-1.6–0.1)			

^‡^ Primary study objective; * Wilcoxon test; ^†^ Mann-Whitney U test.

SD: standard deviation; MCC: maximum cystometric capacity; Pdet_max_: maximum detrusor pressure; QoL: quality of life; SIUP: Specific Impact of Urinary Problems.

### Non-responders and second-line therapies

Eight patients (23.5%) of the Oxy Group and four (11.8%) of the OnabotA Group did not respond to the treatment. Oxy non-responders received 300U OnabotA injections and all showed a good response. OnabotA non-responders were treated with a second OnabotA injection of 300U after which two patients (50%) presented a good response. Two non-responding patients (50%) underwent enterocystoplasty.

### Adverse effects

Macroscopic hematuria was present during the first 24 hours in eight (28%) OnabotA patients. Dry mouth was the most commonly reported adverse event in the Oxy Group (72%), but only one (2.9%) patient discontinued the study for this reason. Eight (23.5%) patients reported a worsening of constipation. No systemic adverse events with OnabotA were reported.

## DISCUSSION

This is the first study to have randomized NDO patients who were treated either with oral Oxy or OnabotA injections. The study fulfilled all quality criteria of a randomized, controlled trial and was adequately powered to compare the groups. Our prospective investigation showed that urodynamic (MCC, Pdet_max_ and bladder compliance), clinical (incontinence episodes/24h) and quality of life parameters significantly improved from baseline to study end (24 weeks), with both Oxy and OnabotA. However, improvement was significantly greater with intradetrusor OnabotA injections. The responder rate was also higher with OnabotA (88.2%) compared with Oxy (76.5%). Both treatments were safe but the adverse events profile seemed to be in favor of OnabotA. Short-term macroscopic hematuria after intradetrusor OnabotA injections in 28% of patients had to be balanced against long-term dry mouth in 72% and constipation in 23.5% of Oxy patients.

Urodynamic studies, with or without active treatment, in patients with NDO due to SCI are scarce. Stöhrer et al.,^(^ ^15^ ^)^ investigated 131 NDO patients (122 with SCI) who were treated with Oxy 15mg/day over a 3-week period. Similar to the present study, the investigators reported a significant improvement of urinary incontinence, MCC, bladder compliance, Pdet_max_, and quality of life. Homma et al.,^(^ ^16^ ^)^ reported that, although tolterodine had a lower rate of adverse events, Oxy was superior to placebo in improving quality of life. In the present study, 2.9% of patients treated with Oxy were unable to tolerate dry mouth. Yarker et al.,^(^ ^17^ ^)^ reported about a discontinuation rate of 25% caused by adverse events due to orally administered, immediate-release Oxy.

Immediate-release Oxy was chosen as a comparator to OnabotA since several controlled studies showed this drug is effective for the treatment of NDO^(^ ^18^ ^)^ and, additionally, Oxy is the only antimuscarinic drug reimbursed by the Brazilian public healthcare system. Oral Oxy is also the only available drug for this indication in most countries, whereas other antimuscarinics are too expensive or have not been globally approved. On the other hand, intradetrusor OnabotA injections are reimbursed in Brazil as second-line treatment of NDO. The 300U dose of OnabotA was chosen because it was often used to treat NDO at the time of patient recruitment. Patients allocated to the 300U OnabotA Group demonstrated a significant improvement of all evaluated urodynamic parameters; therefore, our results are comparable to those published by Reitz et al.,^(^ ^19^ ^)^ However, Cruz et al.,^(^ ^20^ ^)^ and Ginsberg et al.,^(^ ^21^ ^)^ lately reported that no clinically relevant benefit exists for the 300U over the 200U dose of OnabotA with regard to efficacy or duration of effects.

We also observed a significant reduction of the number of urinary incontinence episodes per 24 hours in the OnabotA-treated group; thus, we could confirm results of the two largest randomized-controlled trials published.^(^ ^19^ ^,^ ^20^ ^)^ The OnabotA patients of our study also experienced a significant improvement of quality of life. Schurch et al.,^(^ ^22^ ^)^ reported an improvement in quality of life in 59 patients who were allocated to three groups and treated with 300U or 200U of OnabotA or placebo, and then followed-up for 24 weeks. This study demonstrated a significant improvement of quality of life scores in patients treated with OnabotA, as compared to placebo.

Comparison of the mean differences between the two groups in urodynamic parameters, from baseline to 24 weeks, demonstrated that OnabotA injection therapy was superior to Oxy with regard to all parameters analyzed. The improved urodynamic parameters resulted in a higher continence rate and, secondarily, in a greater impact on quality of life. According to Pannek et al.*,* ^(^ ^23^ ^)^ treatment regimens resulting in improved urodynamic outcome and urinary continence are associated with quality of life improvement. We believe that in our study another factor contributed to improved quality of life in the OnabotA Group, *i.e*., the better tolerability profile since no long-term adverse events were reported. Dry mouth was the adverse event most often described by patients treated with Oxy (72%) in our study, compared with 17 to 97% of patients receiving immediate-release oral Oxy in the literature.^(^ ^24^ ^)^ Mild macroscopic hematuria during the first 24 hours after intradetrusor OnabotA injection was found in eight (28%) patients, in comparison with the incidence rate of 2 to 21% reported by Karsenty et al.,^(^ ^3^ ^)^ Autonomic dysreflexia during urodynamic investigation or bladder filling for OnabotA injections could be a potential threat especially in SCI patients with a complete lesion above T6.^(^ ^25^ ^)^

Urinary continence rates in our study were significantly higher in patients with OnabotA (60%) compared with Oxy (6%). Karsenty et al.,^(^ ^3^ ^)^ found urinary continence ranging from 40 to 80%; therefore, our results are in accordance with those previously published. SCI experts agree that achieving continence and decrease in Pdet_max_ (<40cmH_2_O) are two key components of NDO management.

In four patients (14%) treated with OnabotA, no changes were found in the urodynamic parameters or quality of life scores, and one patient presented with a reduction in bladder compliance. Reitz et al.^(^ ^19^ ^)^ identified 9 out of 200 patients (4.5%) who experienced no clinical or urodynamic benefit after OnabotA injections. The reasons for the lack of response in these patients remains unknown, but mistakes made during dilution or intradetrusor injection of OnabotA cannot be excluded.

Although our study had the highest quality standards, there are also limitations. Immediate-release Oxy is associated with the highest rates of adverse events of all antimuscarinics, although dry mouth has also been reported to be the most frequent adverse event with any other antimuscarinic agent. Moreover, immediate-release Oxy is the only oral drug available for the treatment of NDO in the Brazilian public healthcare system and also in many other countries. We compared only two different modalities for the treatment of NDO but future studies need to compare OnabotA injections with other antimuscarinics drugs. Blinding of patients with placebo (in the Oxy Group) or sham treatment (in the OnabotA Group) would have been desirable but was not granted by the local ethic committee to avoid the risk of upper urinary tract damage and guarantee active treatment.

## CONCLUSION

The comparison of both objective (bladder diary and urodynamic data) and subjective (quality of life questionnaire) parameters, onabotulinumtoxinA injections were found to be significantly more efficacious than oral oxybutynin. Due to the better results of onabotulinumtoxinA we should consider this treatment as the first line option in neurogenic detrusor overactivity patients when it is only possible to use oxybutynin.
